# Cell–cell fusion as a potential target in cancer therapy

**DOI:** 10.3332/ecancer.2009.145

**Published:** 2009-08-06

**Authors:** JM Gasent Blesa, VA Candel

**Affiliations:** 1 Hospital General Universitari Marina Alta, Denia, Plana de l’Est no 5, Alacant, Spain; 2 Hospital Universitari Arnau de Vilanova, Valencia, Spain

## Abstract

In the fight against cancer, new and more specific targets are needed. Here, we offer an example of a potential target that has not been widely studied, namely the syncytin protein. Syncytin is expressed mainly in the human placenta and is implicated in placental syncytiotrophoblast cell fusion. Not much is known about the role of syncytin in cancer, but the existing data call for more intense research. Its retroviral origin and particular tissue distribution make syncytin an interesting potential target in cancer therapy.

## Translational relevance

This paper reviews an unexplored potential new target in cancer therapy. We consider the origin of syncytin, its normal role in human placentogenesis and data regarding its relevance in cell–cell fusion. Given its particular distribution in normal human tissues and its potential roll as an immune modulator and in amino acid transport, tissue tolerance and protection against retroviral infection, syncytin could be a potential target for immune therapy using specific inhibitors. Current data about the role of syncytin in cancer are also discussed.

## Introduction

Transposable elements (TEs) or mobile elements are genetic components that can have a significant effect on the host genome [1,2]. Furthermore, these elements form the majority of the human genome. Insertions of TEs influence the transcriptional regulation of some genes as well as the termination of transcription. Hundreds of examples have been identified in which individual exons possess sequences that are similar or identical to TE fragments [3,4]. In many of these cases, a single exon is involved, and its transcription yields a variant mRNA [5]. It has been suggested that TEs are a source of variation as a result of the insertion of fragments of their sequence into functioning genes elsewhere in the genome.

One of the potential effects of TEs is the generation of new gene sequences [3,6,7] such as the envelope (env) genes of retroviral origin found in several mammals. In particular, the human genome contains full-length copies of four env open-reading frames (ORFs) that are highly expressed in the placenta [8–10].

The best known of the human env-derived genes is syncytin-1, or ERVWE1, which originated from a human endogenous retrovirus (HERV) of the HERV-W family inserted on chromosome 7q21 in human-pre-hominoids 40–45 million years ago [11,10]. The env gene maintained an ORF coding for a 538-amino acid polypeptide that has all the characteristic features of env proteins and mediates intercellular fusion *in vitro* [11–13].

Recently, a molecular evolution study of the HERV-W provirus in several ape species and 24 humans demonstrated the conservation of syncytin-1, as well as its retention of receptor-mediated fusogenic activity [14]. However, an analysis of the synonymous and non-synonymous substitutions indicated a relatively high degree of amino acid change in hominoids [15], which could be consistent with a low degree of selective pressure. HERV-W invasion of the primate genome has been documented as occurring before the divergence of hominoids and Old World monkeys [16,17] and as being inactive [18].

Another syncytin, syncytin-2, has also been identified; it is functional in all primates, including New World monkeys, but it is absent from prosimians, thus dating its entry into the primate genome to about 40 million years ago [19,20]. The syncytin-2 gene encodes a 529-kDa polypeptide with a general organization similar to that of retroviral envelopes: a cleavage site separates the surface subunit that binds a still unidentified receptor on the target cell from the transmembrane subunit (TM), which is classically thought to be involved in the fusion process [21]. The TM subunit harbours an N-terminal fusion peptide, an ectodomain with a leucine zipper motif, a highly hydrophobic transmembrane anchor and a C-terminal intracellular domain.

The ectodomains of retroviral envelopes are believed to play a pivotal role in their fusogenic function. Indeed, by analogy with the pre-fusion and post-fusion structures of influenza haemagglutinin, they include a short loop that undergoes a dramatic change in geometry during the fusion process [22,23] switching the ectodomain from an entirely a-helical to a helix-turn-helix conformation. When the structure of the syncytin-2 central domain is compared to that of present-day retroviruses, such as MoMLV and the human T-cell lymphotropic virus (HTLV-I), the geometries of the pivotal loops of all three ectodomains appear virtually identical, despite completely different amino acid sequences [24].

## Functions of syncytin

The HERV-derived syncytin may have played a role in the evolution of the human placenta due to its niche function in human placentogenesis, albeit in a highly specific manner, as the placentas of different mammalian species exhibit structural discrepancies [25].

**Figure 1:**
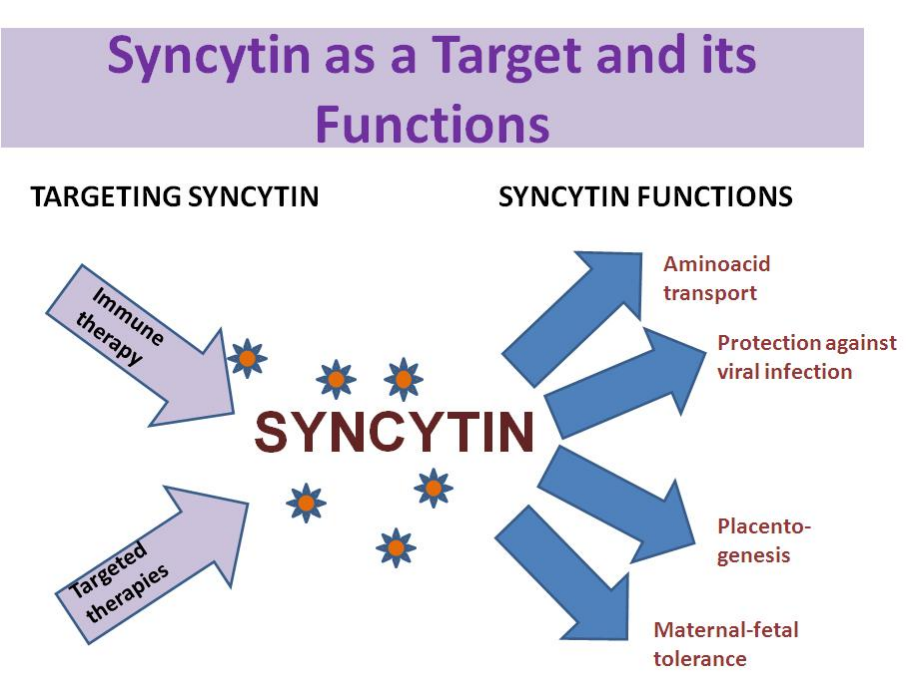
Potential functions of syncytin—targeting syncitin.

Because of its interaction with a specific receptor known to function as a retrovirus receptor and an amino acid transporter, and its stimulation of cell–cell fusion processes, the protein was designated as syncytin (Figure 1).

The syncytin receptor, or at least the first known functional syncytin-binding site, is the sodium-dependent neutral amino acid transporter ASCT2, which transports alanine, serine and cysteine. Elevated syncytin expression is followed by further cell differentiation and generation of the syncytium, formation of gap junctions and an increase in β-hCG secretion [26]. The effects of syncytin can be blocked *in vitro* by antibodies directed against syncytin or by the use of syncytin antisense strategies [11,26]. In addition to *in vitro* studies, it was recently demonstrated that the syncytin locus is strongly preserved in a large cohort of individuals, including long-terminal repeat (LTR) elements involved in the regulation of gene transcription [14]. Apart from these findings, orthologous syncytin loci have been identified in the genomes of great apes [14]; moreover, expression of syncytin has been described in the rhesus monkey endometrium, where it may play a role in decasualization or receptivity [27].

ASCT2 expression has been demonstrated predominantly at the basal membrane of the human syncytiotrophoblast [28–31], which may function as the interaction site between syncytin and its receptor. The observation that syncytin and ASCT2 levels are inversely correlated during cell syncytialization could be partially explained by the phenomenon that retroviruses may induce down-regulation of their receptors [32]. This can be observed after infection of cells with wild-type viruses of the type-D interference group, which impairs neutral amino acid transport [33].

It has been previously reported that an alteration in particular placental amino acid transporters is associated with intrauterine growth restriction (IUGR) and distinctive anthropometric features in the foetus, especially for system A, which transports neutral amino acids [34–36].

System A, in contrast to the ASCT2 transporter, is down-regulated in the case of oxygen deficiency and up-regulated in the case of excess oxygen [28], which may contribute to foetal IUGR. Other transport systems, for example those for arginine, have been mentioned in the context of pre-eclamptic placentas [37].

Another important point concerning syncytin is its potential role in maternal immune tolerance to the foetus. Exogenous retroviral envelope proteins can inhibit the immune responses of leukocytes by means of a highly consistent amino acid sequence [38]. This sequence in the syncytin protein may therefore contribute to a specific immune modulating mechanism within the developing placenta [11]. The nature of additional interactions with other mechanisms such as the MHC class I molecules, in particular, HLA-G, which is thought to produce immune tolerance [39], or immunoregulatory cytokines such as immunosuppressive interleukin-10 [40] remains unclear, although syncytiotrophoblast formation stimulated by syncytin may subsequently ensure syncytiotrophoblast survival via additional interactions with immune or apoptotic mechanisms. Whether, or to what extent, syncytin contributes to maternal immune tolerance of the foetus or interacts with other mechanisms such as apoptotic events at the maternal–foetal interface is currently unclear.

Syncytin expression has been shown to cause cellular resistance to infection by spleen necrosis virus [41], and it is likely to prevent infection by other retroviruses of the same family. It is thus feasible that expression of syncytin by the syncytiotrophoblast prevents vertical transmission of a number of retroviruses through the placenta.

## Gene promoter: description, mutations and repercussions. Fusion in cell lines

The major transcription initiation site of syncytin-1 gene is 56 base pairs (bp) downstream from a putative CCAAT box [42]. Deletion analysis performed on the 59-bp flanking region of the syncytin gene indicated that the proximal 148 bp are essential for minimal promoter activity and that specific regions of the promoter from nucleotides (nts) −1519 to −984 and nts −294 to −148 are required for maximal expression in normal trophoblast cells. DNAse-I footprint analysis of the region between nts −252 and +110 revealed three protected regions, FP1–FP3. Mutagenesis of a hepatocyte-specific nuclear protein-1-binding site in FP1 and a TATA box in FP3 had no effect on basal promoter activity. On the other hand, mutation of the CCAAT motif and the octamer protein binding site in FP2 decreased promoter activity by 88% and 76%, respectively. Mutation of the ecdysone receptor response element in FP2, which may bind a nuclear hormone receptor, increased basal promoter activity. Gel shift and supershift assays indicated that the CCAAT-binding factor binds to the CCAAT motif and that octamer binds to the octamer-binding site. Thus, it can been deduced that the syncytin promoter is located in the 59-bp-long terminal repeat of the HERV-W gene and that binding sites for GATA and Oct in the proximal promoter are critical for transcriptional regulation of syncytin in trophoblast cells [42].

The 146-bp stretch of the 59-bp flanking region in the human syncytin gene from nts 294 to 148 is essential for basal gene expression in human BeWo and JEG3 choriocarcinoma cell lines, but not in hepatoblastoma and kidney cell lines. Ligation of the 146-bp fragment to an SV40 (Simian Virus 40) promoter or a human β-globin minimal promoter markedly enhanced promoter activity in placental cells, but not in liver or kidney cells. DNase-I footprint assays indicated that nuclear extracts from BeWo cells, but not HepG2 cells, protected four regions (FP1–FP4) of the 146-bp fragment. Furthermore, site-directed mutagenesis of an SP1-binding site in FP3 and a GATA-binding site in FP4 significantly repressed promoter activity in the placental cells. Over-expression of SP1 (Sp1 transcription factor), GATA2 (GATA binding protein 2) and GATA3 induced syncytin promoter activity but had little or no effect on the activity of syncytin promoter fragments containing mutations in the SP1- and GATA-binding sites. In addition, GATA2 and GATA3 mRNA levels markedly increased during spontaneous *in vitro* differentiation of human cytotrophoblast cells after they fused to form a syncytium. These findings suggest that the 146-bp region of the 59-bp flanking region (nts 2294/2148) of the human syncytin gene acts as a placenta-specific enhancer. Furthermore, binding of SP1 and GATA family members to this enhancer is critical for cell-specific expression of the syncytin gene [43].

## Fusion in cancer cells

Mortensen *et al* [44] found that human breast cancer cells fused with endothelial cells in culture. Expression of syncytin-1 protein has also been observed in breast cancer cell lines [45], and both tumour cells and endothelial cells express the syncytin-1 receptor ASCT-2. Using syncytin-1 antisense oligonucleotides to down-regulate syncytin-1 expression inhibited breast cancer-endothelial cell fusion. In addition, the syncytin-1 CHR peptide also inhibited cell fusion [45]. Neither the antisense nor the CHR peptide experiments, however, effected total inhibition of cancer-endothelial cell fusion. It is possible that the antisense oligonucleotides did not completely eliminate syncytin-1 expression. On the other hand, syncytin-2 protein expression has also been documented and could contribute to cancer–host cell fusion. Further experiments using shRNA-directed down-regulation of both syncytin-1 and -2 proteins, as well as additional putative fusogenic retroviral sequences, are underway to test this possibility [46].

These data provide strong evidence that syncytin-1 protein is involved in mediating cancer-endothelial cell fusion *in vitro*.

The involvement of syncytin-1 in tumour cell fusion events was subsequently confirmed by Strick *et al* [47] in endometrial carcinomas. In their study, down-regulation of synctytin-1 protein expression also inhibited fusion between endometrial tumour cells. In agreement with findings in placental cells, both cAMP-elevating agents and oestrogens up-regulated syncytin-1 protein expression; however, only cAMP-elevating agents stimulated cell fusion [48]. This apparent discrepancy could be ascribed to the fact that oestrogen treatment up-regulates TGF beta, which interferes with syncytin-induced fusion. Indeed, oestrogen did stimulate cell fusion if TGF beta was neutralized with antibodies. Conversely, addition of TGF beta 1 or 3 reduced the cell fusion induced by cAMP-elevating agents. This effect was observed in both endometrial carcinoma cells and trophoblast-derived cells. These results show that both cAMP and oestrogens may positively regulate syncytin-1 protein expression in tumour cells and that TGF beta family members may negatively regulate the fusogenic effects of syncytin-1 in both trophoblasts and cancer cells [48]. The role of syncytin-1 and cell fusion in cancer requires further study.

Published data show that the syncytin-1 protein is not the only fusogenic protein expressed by breast cancer cells, and the results presented by Strick *et al* [47] show that additional regulators, such as TGF beta isoforms, may be important modulators of cell fusion.

This is not to say that cell fusion may be universally beneficial to all cancers. First, syncytins may have additional effects within the tumour environment [46,47]. Second, it seems likely that several factors within the cancer and its stroma (e.g., inactivated tumour suppressor genes, activated oncogenes and expression of fusogens and of CD9, CD81 and TGF beta) may function together to bring about a tumour profile as diverse as that demonstrated by the cell fusion experiments referenced above. Expression of CD9 has been analysed in a number of tumours and seems, like syncytin expression, to correlate with a good prognosis [48–50].

## Syncytin and endometrial carcinoma

The vast majority of endometrial carcinomas express the oestrogen and progesterone receptors and are thought to develop from endometrial hyperplasia induced by steroid hormone stimulation. Strick *et al* [47] demonstrated that syncytin-1 expression is regulated via an oestrogen response element in these steroid-driven tumours, resulting in increased proliferation of primary endometrial carcinoma cells and cell lines. It is noteworthy that activation of cAMP signalling by forskolin or SP-cAMP also resulted in syncytin-1 gene up-regulation, although this intervention led to increased cell–cell fusion instead of proliferation. Furthermore, they showed that, after stimulation with steroids or cAMP activation, syncytin-1 gene is involved in anchorage-independent colony growth and fusion. The switch between cell proliferation and cell-–cell fusion was shown to be subject to a complex regulatory mechanism involving both steroid hormone- and TGF-β1/TGF-β3-dependent effects. The authors’ findings raise the possibility that syncytins play an important role in the progression of hormone-dependent cancers, such as endometrial carcinoma. In healthy individuals, syncytin-1 protein expression is usually restricted to the placenta, and it has been suggested that CpG hypomethylation of the 5′ LTRs of both syncytin genes is one of the underlying regulatory mechanisms [51]. CpG hypomethylation also appears to be a means of activating syncytin-1 protein expression in ovarian carcinoma [52].

Since syncytin is up=regulated in endometrial carcinoma, the results of Strick *et al* [47] suggest that syncytin-1-mediated cell fusion may promote rather than suppress tumour growth. They demonstrated that syncytin-1 expression and function are highly dependent on hormonal regulation: steroid hormones such as estradiol (E2) induce the expression of syncytin-1 protein and result in increased cell proliferation and anchorage-independent growth. On the other hand, activation of cAMP signalling promotes both endometrial carcinoma cell fusion and anchorage-independent growth. Cell fusion can be blocked by TGF-β1/3, which is itself subject to steroid hormone regulation. Thus, in the presence of steroid hormones, TGF-β provides a switch between syncytin-1-mediated cell fusion and cell proliferation. Oestrogen is a key aetiological factor in endometrial carcinogenesis. Regulation by TGF-β and the involvement of syncytin-1 add a new dimension to our understanding of endometrial carcinoma progression. These findings reveal potential new directions for therapeutic approaches that circumvent the need for potentially risky endocrinological interventions.

The identification of syncytin as a target of TGF-β in endometrial carcinoma not only provides novel insight into the molecular mechanisms of tumour progression but may also have important clinical implications. A peptide capable of inhibiting syncytin-mediated cell–cell fusion was recently shown to reduce breast carcinoma cell fusion with human umbilical vein endothelial cells by 50% (p<0.001) [45]. While this inhibitor has been tested only in an experimental setting and its toxicity profile has not yet been evaluated, the use of this peptide or of related reagents may have beneficial effects in the treatment of endometrial and, possibly, other carcinomas. However, there is a need to evaluate the contribution of cell fusion to the malignant phenotype of endometrial carcinoma cells to rule out the possibility that expression of syncytin is a mere physiological response of the host to the tumour. Such a response could, for example, be aimed at neutralizing malignant cells via fusion with normal cells expressing tumour suppressor genes [53].

## Syncytin and breast cancer

Two series of human breast cancer patients were evaluated for tumoural expression of syncytin-1, using a polyclonal antiserum raised against a synthetic nanopeptide derived from the syncytin-1 sequence. In addition, tumours were also screened for expression of ASCT-2 using a peptide antiserum. Pre-absorption of the antisera with the corresponding peptides, but not with irrelevant peptides, abolished staining [54].

The results show that 38% of all breast cancer samples displayed detectable staining for syncytin and that endothelial cells expressed ASCT-2. Moreover, significant expression of ASCT-2 was detected in many tumour cells. The degree of syncytin immunostaining was visually graded using coded specimens and statistical analysis showed that it correlated positively with the disease-free survival of patients [47]. Multivariate analysis included age dichotomised at 40 years, tumour size dichotomised at 20 mm, grade and adjuvant therapy, and it identified syncytin expression as an independent prognostic indicator of increased disease-free survival. When used as a continuous variable, syncytin expression emerged as a significant prognostic indicator of disease-free survival in the Cox model (**p** = 0.02) [47].

## Conclusions

We believe that syncytins may be important fusogens for both trophoblast and cancer cell fusion, and that they may mediate additional cell fusion events, acting in concert with other molecules that have both enhancing and inhibitory regulatory effects. Both effects are relevant in mammalian placentogenesis. Syncytins have also been implicated in amino acid transport [28], immune tolerance [38] and protection against retroviral infections [41]. These functions of syncytin could all be utilized by cancers to gain a growth advantage, and, in our opinion, these functions should be studied in neoplasias.

Correlations of syncytin with clinical parameters are scarce, but the most relevant data have been reported in breast cancer [53]. Besides laboratory approaches and clinical studies correlating syncytin-1 expression in endometrial carcinoma, breast carcinoma and other tumours, we believe that syncytin should be studied in relation to histopathological and clinicopathological parameters in order to assess the relevance of this potential novel molecular target. Its unique localization and distribution make syncytin attractive for the development of targeted therapies and immune therapies.
